# New 10-Year Atherosclerosis Cardiovascular Disease Risk Score for Asian and American Indian Women Service Members

**DOI:** 10.1016/j.jacasi.2026.01.003

**Published:** 2026-02-27

**Authors:** Haekyung Jeon-Slaughter, Erum Z. Whyne, Callum Doyle, Xiaofei Chen, Sy Han Chiou, Shirling Tsai, Ramin Ebrahimi

**Affiliations:** a VA North Texas Health Care System, Dallas, Texas, USA; b Department of Internal Medicine, University of Texas Southwestern Medical Center, Dallas, Texas, USA; c Department of Statistics and Data Science, Southern Methodist University, Dallas, Texas, USA; d Oncology Biometrics, AstraZeneca, Gaithersburg, Maryland, USA; e Department of Surgery, University of Texas Southwestern Medical Center, Dallas, Texas, USA; f Veterans Affairs Greater Los Angeles Health Care System, Los Angeles, California, USA; g Department of Medicine, University of California at Los Angeles, Los Angeles, California, USA

**Keywords:** Alaska Natives, American Indians, ASCVD risk score, Asian women, cardiovascular disease, Native Americans, women service members, women veterans

## Abstract

**Background:**

To date, there is no validated atherosclerosis cardiovascular disease risk calculator developed for Asian/Hawaiian (Asian) Americans and American Indian/Alaska Native (AI) women service members based on large, representative data.

**Objectives:**

This study developed a validated Department of Veterans Affairs (VA) women cardiovascular disease (CVD) risk score for Asian and AI women service members and veterans aged 20 to 80 using VA and Military Health System electronic health record data, largest electronic health record data in the United States.

**Methods:**

The study extracted data from the VA Corporate Data Warehouse and selected 15,042 unique Asian and 5,663 AI women service members and veterans who received care from the VA and Military Health System between 2007 and 2022. Closely following the model construct of the VA women CVD risk score, a time-varying covariate Cox model was used to estimate effects of CVD predictors—age, untreated and treated systolic blood pressure, antihypertensive medication, total cholesterol, high-density lipoprotein cholesterol, diabetes, active smoking, and major depression—for each race group separately. Internal and cross validation of the models was conducted.

**Results:**

The VA women CVD risk score for Asian and AI women service members showed good model accuracy, with C-statistics of 0.78 and 0.77, respectively. The calibration plots showed approximation to a 45° line—perfect model fit, and internal validation and cross-validation results showed good validation.

**Conclusions:**

The new Asian/AI VA women CVD risk scores showed both good discrimination and accuracy and, thus, are appropriate to assess 10-year atherosclerosis CVD risk for Asian and AI women service members and veterans.

Heart disease is the leading cause of death among women, and women service members have a much higher risk of atherosclerosis cardiovascular disease (ASCVD) events—myocardial infarction, stroke, cardiac arrests, and cardiac deaths— at much younger ages than their civilian counterparts due to military exposure.^1^, ^2^, ^3^ Military exposure includes a range of chemical, physical, and environmental hazards during military service, which are known to be associated with increased ASCVD risk.^4^ Women’s heart disease has often been understated and underestimated because of a lack of adequate assessment tools, especially for women with diverse backgrounds, including Asian American, Hawaiian American, American Indian, and Alaska Native women. This is despite the fact that Asian American is one of the fastest-growing racial/ethnic groups in the United States^5^ and certain Asian subgroups have a greater risk of heart disease at earlier ages than non-Hispanic White individuals. In addition, American Indian and Alaska Native individuals are disproportionately burdened by heart disease with a higher prevalence rate than the general population^6^ and 20% greater fatality from cardiovascular events.^7^ In 2023, Asian women represented 3.0% and American Indian/Alaska Native (AI) women 1.0% of the women veteran population.^8^ In 2022, Asian women represented 6.0% and AI women 1.3% of the active-duty women service member population.^9^ A greater number of Asian American and AI individuals in the general population is also reflected in the active-duty and veteran population.

The Department of Veterans Affairs (VA) women cardiovascular disease (CVD) risk score is one of the first major successful initiatives to provide an accurate CVD risk assessment for women veterans^10^; additional tools for Asian American and American Indian women are much needed. Traditional risk scores (eg, Framingham and Pooled Cohorts Equation) may lead to inaccurate risk estimation^11^ in these populations and have been found to underestimate CVD risk in Asian individuals.^12^ Furthermore, other risk indicators, such as coronary artery calcium scores have found sex differences indicating the need for sex-specific tools in Asian populations.^13^

The VA women CVD risk score was developed by leveraging the large, representative VA electronic health record (EHR) data on women veterans and successfully externally calibrating to the non-VA population—civilians and younger military service members.^14^ The VA women CVD risk score assesses 10-year ASCVD risk of women and includes non-Hispanic (N-H) White, N-H Black, and Hispanic women aged between 20 and 80 years, with major depression as a predictor. Since its publication in 2021,^10^ the VA women CVD risk score has been available as clinical decision support apps/medical calculators, and as recommended CVD clinical toolkits for VA pharmacists and primary care physicians in the Computerized Patient Record System, an EHR system used by the VA, to treat CVD for women veterans.^14^ The VA women CVD risk score is available online.^15^ The predicted ASCVD risk is presented as a numerical result along with its interpretation, that is, CVD risk stratifications—low (<7.5%), moderate (7.5%-19.9%), and high risk (≥20.0%). Factors in the VA women CVD risk score are automatically prefilled from VA Computerized Patient Record System records, and the values can be modified as needed to reduce barriers of use (eg, time constraints, lack of EHR integration, and limited information); however, large buy-in from clinicians (eg, reminders) still poses a challenge.

Although the VA women CVD risk score includes N-H White, N-H Black, and Hispanic women, unlike other existing ASCVD risk scores, such as the 2013 American College of Cardiology/American Heart Association (AHA) ASCVD risk score^16^ and AHA’s PREVENT (American Heart Association Predicting Risk of CVD EVENTs),^17^ currently, there is no validated ASCVD risk score for Asian American and Hawaiian American (henceforth, Asian) or American Indian and Alaska Native (henceforth, AI) women service members and veterans. This is despite the growing number of individuals from these populations in the military, especially post 9/11.^18^ Race-stratified models such as VA women CVD risk score and the pooled cohort American College of Cardiology/AHA ASCVD risk score estimated 10-year ASCVD risk higher than race-free models such as the AHA PREVENT model.^19^

Leveraging the large scale of the national VA and military health care system data, the study developed and validated Asian and AI VA women CVD risk scores using a cohort of women service members and veterans who received care from the Veterans Health Administration and direct care from the Military Health System (MHS). As such, the study’s new Asian and AI VA women CVD risk scores are the first appropriate and validated scores to assess ASCVD risk of Asian and AI women service members and veterans.

## Methods

### The Asian and AI women service members and veterans cohort

The visit records of women service members and veterans treated at the military health care system directly and the VA Health Care System between January 1, 2007, and December 31, 2022, were extracted from the VA national Corporate Data Warehouse (CDW). The CDW is a national data repository that contains data from various sources, including the Veterans Health Administration (eg, veteran data) and Department of Defense (eg, current service members data) and incorporates important clinical data, such as vital signs, disease diagnoses, a history of cigarette smoking, chemistry, microbiology, and pathology lab results, available via the VA Informatics and Computing Infrastructure.

The EHR data on women veterans who received health care from the VA Health Care System are available from CDW, a detailed description is available elsewhere from the previous studies.^10^^,^^20^ In addition, EHR data on women service members treated directly from the MHS are also available from CDW under the Department of Defense and Veterans Affairs Infrastructure for Clinical Intelligence (DaVINCI) data structure. The MHS Data Repository is the most comprehensive source of health care data on active service members and is available via DaVINCI data. The DaVINCI data contain active-duty women service members who receive direct health care at military base hospitals and clinics, as well as those who seek care outside of the MHS, but paid for by the MHS.

**Figure 1:**
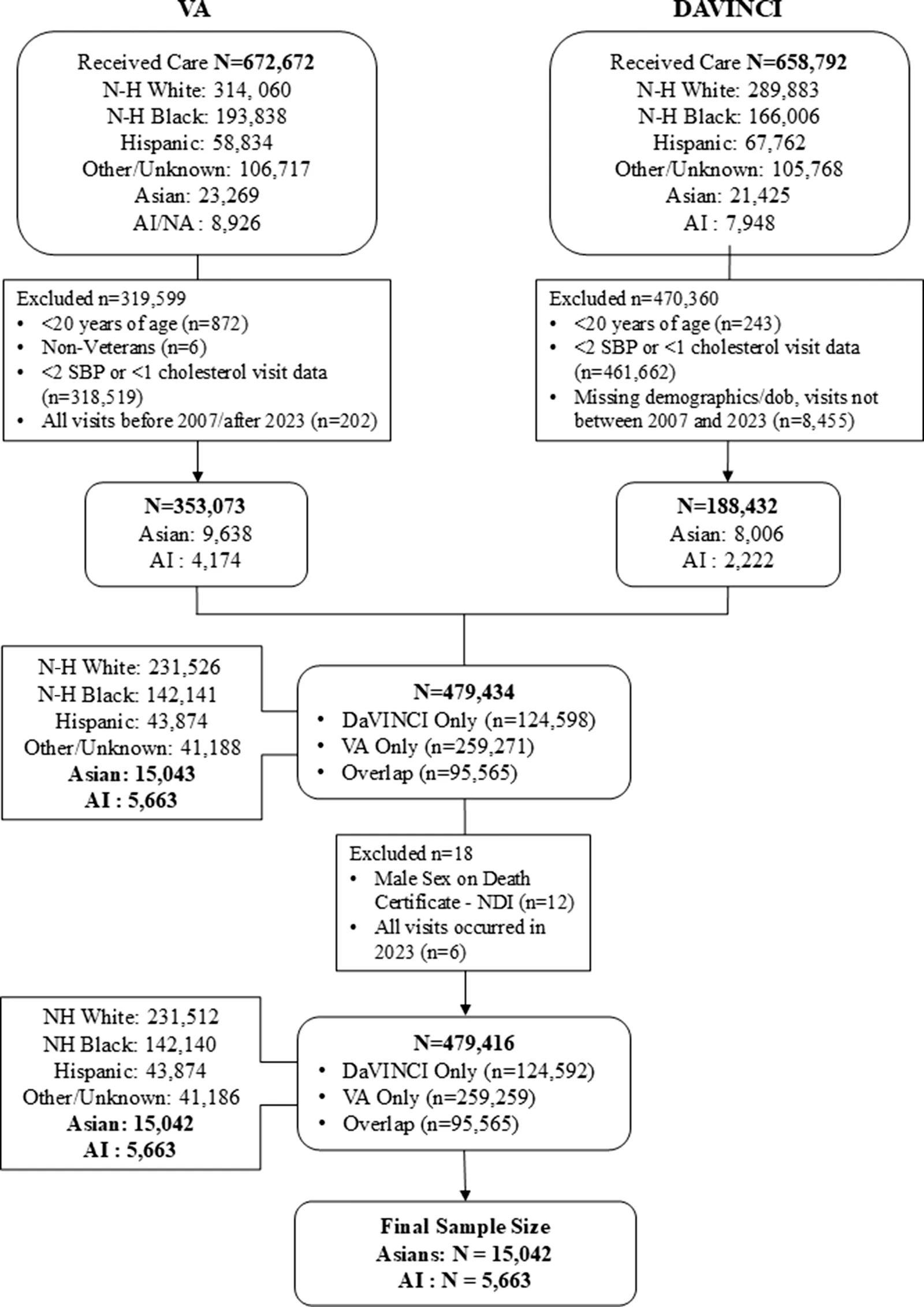
Flowchart of VA Asian and AI Women Cohort A total of 15,042 Asian and 5,633 American Indian and Alaska Native (AI) women who received care from the military and/or VA Health Care System between January 1, 2007, and December 31, 2022, were included in the study cohort. Women were included if: 1) they were between the ages of 20 and 80 years; 2) had 2 or more SBP and 1 or more cholesterol visits; and 3) no known prior (before first visit) cardiovascular disease event. AI = American Indian and Alaska Native; DaVINCI = Department of Defense and Veterans Affairs Infrastructure; NA = Alaska Native; N-H = non-Hispanic; SBP = systolic blood pressure; VA = Department of Veterans Affairs.

The study selected N-H Asian, with background of Asian and Hawaiian Americans (Asian), and American Indian/Indigenous Americans and Alaska Native (AI) women who were between the ages of 20 and 80 years and alive on January 1, 2007, and had no known prior CVD events as a cohort. The current age range, 20 to 80 years, was chosen to include younger active military women service members. The study cohort was then refined to women service members who had complete data on demographics (eg, sex, date of birth), blood pressure measurements (at least 2),^21^ and lipid panel tests (at least 1).^22^^,^^23^ The final set yielded 15,042 Asian women and 5,663 AI women service members and veterans, which represented 3.1% and 1.2% of the total women veteran and service members sample, respectively. Our study cohort broadly reflects the population of Asian and AI women veterans and service members. Figure 1 depicts the inclusion and exclusion flowchart yielding the final sample size of the Asian and AI women service member and veteran cohorts.

The study used the national death index to adjudicate death events and identified cause of death of those who were deceased during the study period. The current study used military enrollment records, the Defense Enrollment Eligibility Reporting System, to specify Asian and AI groups.

### Variables of interest and model construct

The Supplemental Methods section describes the data construct and handling of missing data.^23^ The model constructs include age, untreated and treated systolic blood pressure (SBP), antihypertensive medication treatment, total cholesterol, high-density lipoprotein cholesterol (HDL-C), diabetes mellitus (DM), current smoking status, and major depression, the same model predictors used for the VA women CVD risk score (see Supplemental Methods, section 3. Variable construct). The outcome variable is ASCVD events—nonfatal myocardial infarction, nonfatal hematologic and ischemic strokes, nonfatal cardiac arrests, and cardiac deaths. Age, untreated and treated SBP, total cholesterol, and HDL-C were log transformed to preserve normality. Having ASCVD events, presence of DM, and major depression diagnoses were constructed using International Classification of Diseases (ICD)-9th Revision (ICD-9) and -10th Revision (ICD-10) diagnosis and procedure codes extracted from VA CDW data. The ICD codes to define each variable were validated using available documentation from the VA Phenomics Library. The VA Phenomics Library compiled VA EHR-based phenotype algorithms including ICD codes to define diseases. Chen et al^20^ described detailed ICD codes used to construct each risk factor included in the CVD risk score calculation and validation of ICD codes used to construct CVD events with providers' notes. Construct of current smoking status variable extract data was from VA CDW Health Factor type (smoking status) data available within the VA EHR using natural language process (details available from Chen et al^20^) (Supplemental Material).

### Model estimation and statistical methods

Model estimation in predicting 10-year ASCVD risk closely followed Chen et al^20^ and Jeon-Slaughter et al.^10^ Stratified by race group, a time-varying covariate Cox model with individual random effects was used to estimate coefficients of predictors (Supplemental Figures 1 and 2). Data on factors were repeatedly measured from multiple visits during the study period. Age, SBP, total cholesterol, and HDL-C were natural log transformed to follow log normal distributions. Each VA woman had CVD risk factors measured repeatedly from multiple visits during the study period. The time-variant covariate Cox model assumes that effects of time-varying factors on CVD risk follow step functions.^10^ The predicted 10-year ASCVD risk score is calculated as 1−${S\left(10\right)}^{e^{x\hat{\beta }-\bar{x}\hat{\beta }}}$, where *S(10)* is the 10-year CVD event-free survival probability, *x* is a vector of covariates in the model, $\bar{x}$ is the mean value of corresponding covariates, and $\hat{\beta }$ is a vector of risk coefficients corresponding covariates. A 95% CI of the predicted 10-year ASCVD risk score is calculated using the formula, risk score ± 1.96 · SE (risk score), where SE (risk score) = sqrt( x ⋅ cov($\hat{\beta }$ ˆ) ⋅ x^T^)) where cov($\hat{\beta }$ β ˆ) is a variance-covariance matrix of estimated coefficients ($\hat{\beta }\text{,}$Supplemental Tables 1 and 2 for Asian and AI women, respectively), *x* is a column vector of all covariates (9x1) and *x* ^T^ a transpose of a vector of *x*. 1.96 is a *z* score of a standard normal distribution at α = 0.05 for a 2-sided test.

Model accuracy in discriminating ASCVD events from no events was examined using Harrell C-statistics, and model accuracy predicting ASCVD events was examined by calibration plots.^24^ Harrell C-statistics measure concordance of the model,^13^ C-statistics ≥0.7 represent good model discrimination, a 45° line in a calibration plot represents perfect agreement between predicted and observed ASCVD event probabilities, and the calibration intercept “0” and slope “1” represent a perfect agreement^25^ (for detailed description please see Jeon-Slaughter et al^19^). Proper statistics such as Brier score (in which “0” represents a perfect fit) were presented for model accuracy.

Internal validation of the model predictability was conducted to evaluate the stability of the new Asian and AI VA women CVD risk model coefficient estimates by applying 2 internal validation methods, bootstrapping and cross-validation. The study used a bootstrap method to resample from the women service member cohort data with replacement and the same sample size. We repeated this 100 times and averaged all estimates (bias and SE deviation). The study also conducted a 10-fold cross-validation. This method randomly draws 90% of women service members from the cohort for model development (training data) and uses the remaining 10% for a model validation (validation data). This procedure is repeated 10 times to obtain average estimates. The optimism of the performance (value of C-statistics bias) was used to evaluate internal and cross-validation. Ideally, optimism should be close to 0; thus, the bootstrapped optimism-adjusted C-statistics (C-statistics optimism) should be identical to the original C-statistics.

### Net reclassification index

Closely following Jeon-Slaughter et al,^10^ ASCVD risk was classified into low, moderate, and high, and corresponding risk score ranges were <7.5%, 7.5% to 19.9%, and 20% and higher, respectively.^10^^,^^19^^,^^26^^,^^27^ Upward reclassification represents raising a grade of ASCVD risk, for example, from low risk reclassified to moderate or from low or moderate risk reclassified to high risk, whereas downward reclassification represents lowering a ASCVD risk grade. The net reclassification index (NRI) is a sum of the NRI for those who experienced ASCVD events (NRIevent) and those with no events (NRIno event). NRIevent is calculated as: P (predicted risk estimated upward/ASCVD event) − P (predicted risk estimated downward/ASCVD event) and NRIno event as: P (predicted risk estimated downward/no event) − P (predicted risk estimated upward/no event).^26^ Means and 95% CIs of the NRI were calculated using a bootstrap method. A positive value of the NRI indicates improvement of risk prediction performance.

The study was approved by both the VA North Texas Health Care System Institutional Review Board and University of Texas Southwestern Medical Center Institutional Review Board. Obtaining informed consent from subjects was waived.

Structured Query Language (SQL) Server Management Studio (version 2022, Microsoft Corp) was used for data extraction from VA national CDW data, and statistical and graphical analyses were conducted using SAS Enterprise (version 8.3, SAS Institute) and R (version 4.4.1), respectively.

## Results

Asian and AI women service members and veterans represent about 4.3% of women service members and veteran patients treated at VA health and military health systems between January 1, 2007, and December 31, 2022 (Figure 1).

The average ages of Asian and AI women in the development cohort were 34.9 years and 36.4 years, respectively. AI women service members had significantly higher prevalences of diabetes conditions, active smoking, major depression, and antihypertensive medication intake at baseline than their Asian counterparts (Table 1). Although total cholesterol levels were similar between the 2 groups, AI women service members showed significantly higher SBP and lower HDL-C levels than Asian women at baseline. ASCVD event rates over 10 years were significantly higher in the AI group compared with the Asian group (5.1% vs 3.8%, *P* < 0.0001) (Table 2).

**Table 1:** Baseline Predictors by Non-Hispanic Asian and AI Group

	Non-Hispanic Asian (n = 15,042)	AI (n = 5,663)	*P* Value^a^
Age, y	34.91 ± 11.46	36.40 ± 12.43	<0.0001
SBP, mm Hg	119.35 ± 14.40	121.09 ± 14.16	<0.0001
Diabetes	392 (2.61)	187 (3.30)	<0.0001
Current smoking	2,042 (13.58)	1,119 (19.76)	<0.0001
Major depression	1,198 (7.96)	713 (12.59)	<0.0001
Total cholesterol, mg/dL	190.36 ± 38.32	190.90 ± 38.73	0.3684
HDL, mg/dL	55.66 ± 15.11	53.23 ± 14.90	<0.0001
Antihypertensive medication	788 (5.24)	389 (6.87)	<0.0001

Values are n (%) or mean ± SD.

AI = American Indian and Alaska Native; HDL = high-density lipoprotein; SBP = systolic blood pressure.

a Student’s *t*-tests and chi-square statistics were used to test group differences for continuous and binary variables, respectively.

**Table 2:** ASCVD Events Stratified by Asian and AI Groups

ASCVD Events^a^	Non-Hispanic Asian (n = 15,042)	AI (n = 5,663)	*P* Value
All	573 (3.81)	291 (5.14)	<0.0001
Nonfatal myocardial infarction	256 (1.70)	121 (2.14)	<0.0001
Nonfatal stroke	284 (1.89)	163 (2.88)	<0.0001
Nonfatal cardiac arrest	11 (0.07)	3 (0.05)	0.2526
Cardiac death	63 (0.42)	34 (0.60)	0.0881

Values are n (%).

ASCVD = atherosclerosis cardiovascular disease; other abbreviation as in Table 1.

a The same patient can experience multiple ASCVD events.

**Figure 2:**
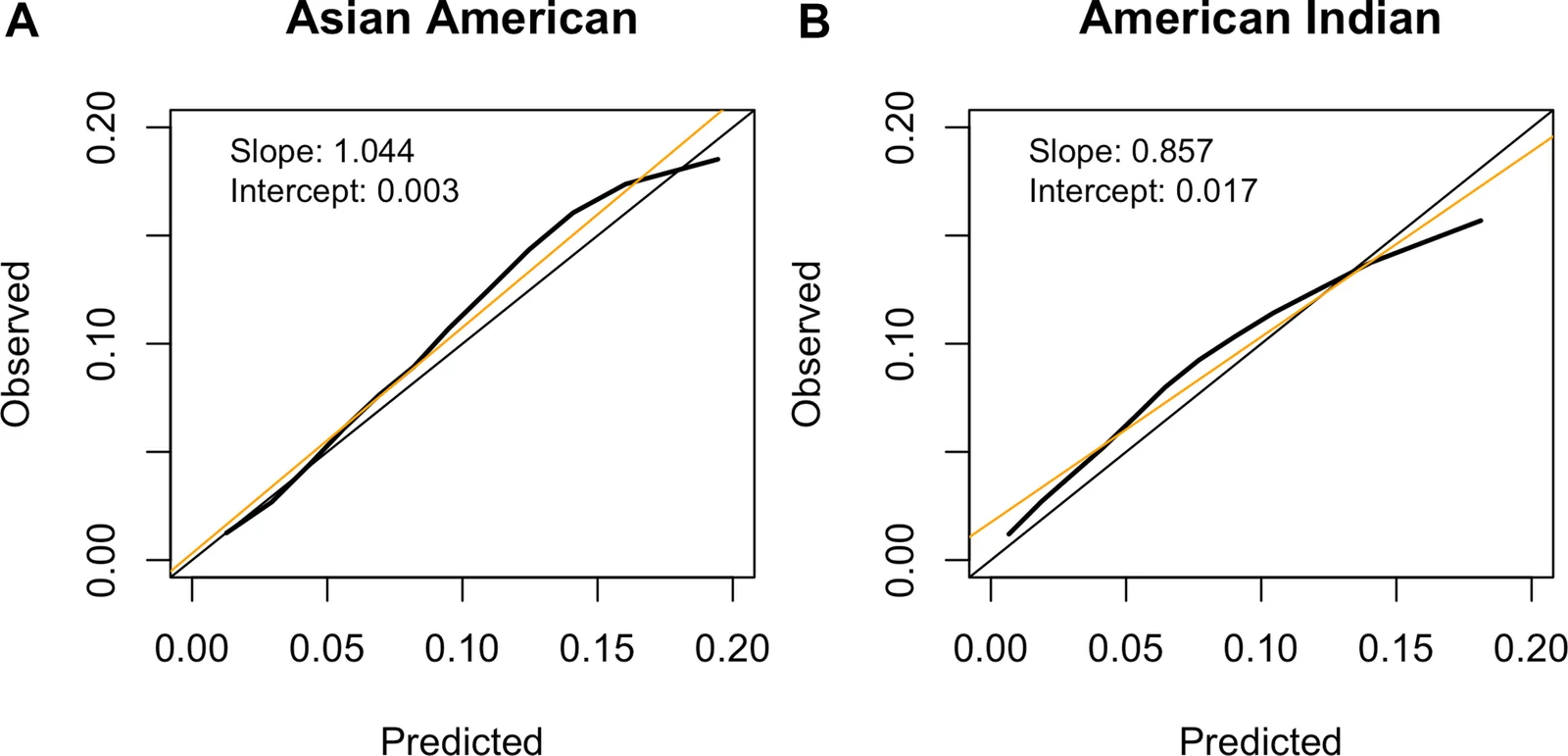
Calibration Plots: Asian and AI Women Service Members and Veterans Calibration plots of 10-year ASCVD risk for Asian service members and veterans (A) and AI women service members and veterans (B) were visualized by plotting predicted probability (x-axis) against observed (y-axis). A 45° line represents perfect model accuracy where the predicted and observed probabilities are the same. The closer the plots are to a 45° line, the better is the accuracy. Brier scores are 0.00002 and 0.00006 for Asian and AI women military service members, respectively. Brier score close to “0” indicates a perfect fit (observed probability = predicted probability). AI = American Indian and Alaska Native; ASCVD = atherosclerosis cardiovascular disease; Asian = Asian American or Hawaiian American.

Table 3 shows the model estimates of the Asian and AI VA women CVD risk scores and Harrell’s C-statistics were 0.78 and 0.77, respectively. Figures 2A and 2B are calibration plots of Asian and AI VA women CVD risk models, respectively. Both calibration plots are very close to a 45° line (intercept 0.001, slope 1.09) (Figure 2A; intercept 0.003, slope 0.96) (Figure 2B), which indicates a good agreement between observed and predicted probabilities of CVD events. Brier scores are 0.00002 and 0.00006 for Asian and AI women military service members, respectively. Tables 4 and 5 show interval validation and cross-validation results, both validation results showed bias close to “0” and small values of root mean squared error. Optimism-adjusted C-statistics for both Asian and AI VA women CVD risk scores were close to “0.”

**Table 3:** Model Estimates Using VA Asian and AI Women Cohorts

	Asian		AI	
	Estimate	SE	Estimate	SE
Ln Age	2.662	0.214	2.540	0.314
Untreated SBP	0.368	0.616	1.311	1.245
Treated SBP	0.266	0.888	−2.247	1.234
DM	0.057	0.126	0.266	0.168
Current smoking	−0.324	0.165	0.127	0.172
Major depression	0.220	0.117	0.057	0.147
Ln total cholesterol	0.313	0.264	0.009	0.367
Ln HDL	−0.939	0.200	−0.560	0.297
Antihypertensive treatment	−0.434	4.305	1.174	6.030
C-statistics	0.776	0.011	0.773	0.015

Asian = Asian Americans or Hawaiian Americans; CVD = cardiovascular disease; DM = diabetes mellitus; HDL = high-density lipoprotein; Ln = natural logarithm; VA = Department of Veterans Affairs; other abbreviations as in Tables 1 and 2.

**Central Illustration:**
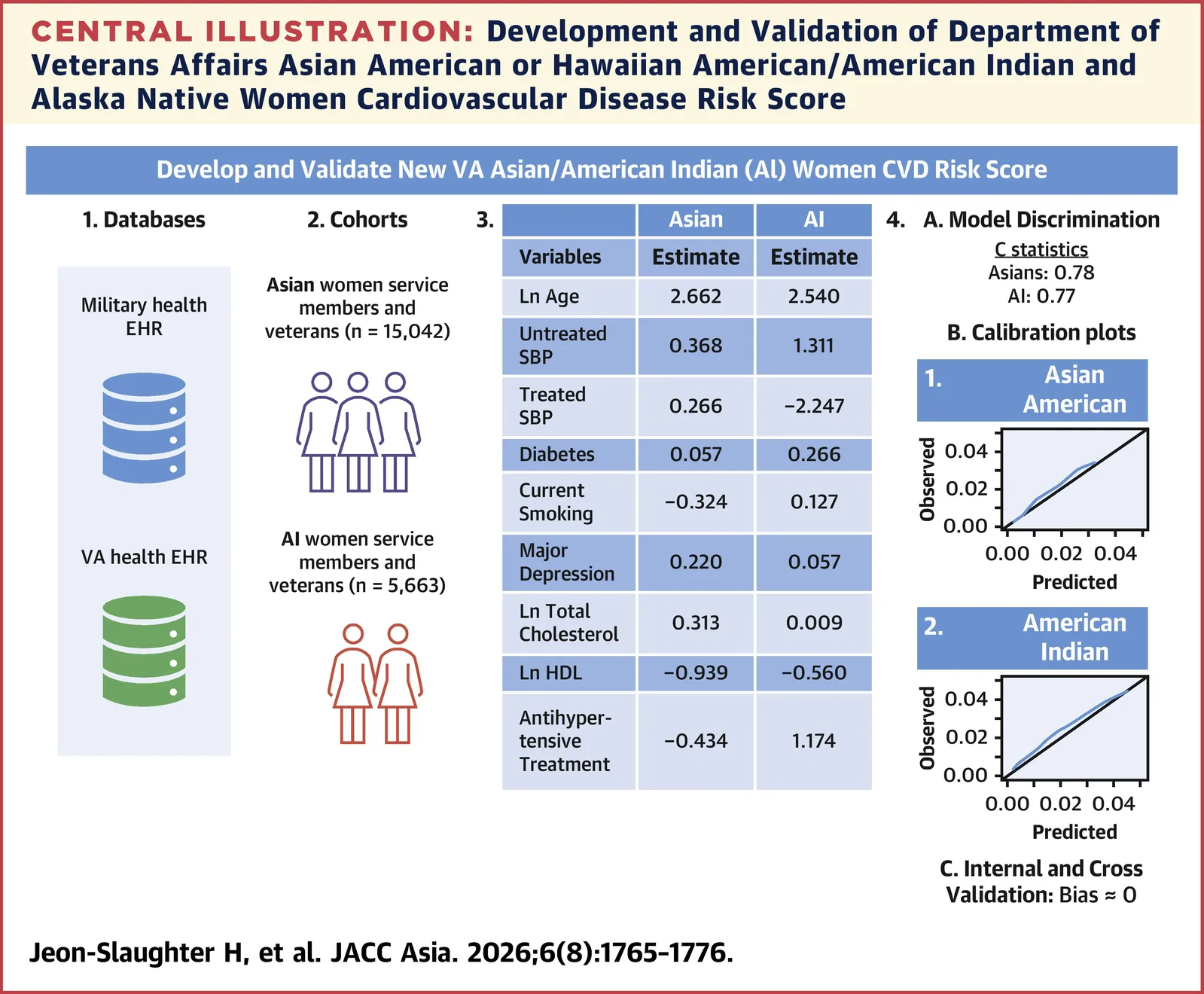
Development and Validation of Department of Veterans Affairs Asian American or Hawaiian American/American Indian and Alaska Native Women Cardiovascular Disease Risk Score This study developed and internally validated the new VA Asian and AI women CVD risk score using data from both the military and VA health system EHR (1. Databases). The study cohort included 15,042 Asian and 5,663 AI women (2. Cohorts). Risk coefficient estimates for variables in model predicting 10-year atherosclerosis cardiovascular disease are presented in panel 3. The model had good discrimination using C-statistics (4A) and calibration plots (4B). A 45-degree line represents perfect model accuracy where predicted and observed probabilities are the same. The closer the plots (4B1 and 4B2) are to a 45-degree line, the better the accuracy. AI = American Indian and Alaska Native; Asian = Asian American or Hawaiian American; CVD = cardiovascular disease; EHR = electronic health record; HDL = high-density lipoprotein; Ln = natural logarithm, SBP = systolic blood pressure; VA = Department of Veterans Affairs.

**Table 4:** Internal Validation (Bootstrap, n = 100) of VA Women CVD Risk Model

Variable	Asian		AI	
	Bias	RMSE	Bias	RMSE
Ln age	0.0358	0.2165	0.0078	0.3599
Ln SBP	−0.0033	0.6545	−0.2072	1.2740
DM	0.0009	0.1197	−0.0246	0.1800
Current smoker	−0.0088	0.1528	−0.0033	0.1617
Ln total cholesterol	−0.0544	0.2583	−0.0086	0.3378
Ln HDL	0.0284	0.1779	−0.0023	0.3136
Major depression	−0.0066	0.1105	−0.0111	0.1577
SBP treatment	−0.1588	4.7827	−1.0983	7.0033
Treated SBP	0.0343	0.9842	0.228	1.4473
Average C-statistics	0.0036	0.0119	0.0022	0.0159

RMSE = root mean square error; other abbreviations as in Tables 1, 2, and 3.

**Table 5:** Cross Validation (10-fold) of VA Women CVD Risk Model

	Asian		AI	
	Bias	RMSE	Bias	RMSE
Ln age	0.0409	0.6423	−0.1170	1.0478
Ln SBP	−0.1215	1.9344	0.5954	4.2826
DM	0.0226	0.3937	0.2352	2.0152
Current smoker	0.0681	3.0864	0.4961	2.6274
Ln total cholesterol	0.0305	0.8445	0.0400	1.0169
Ln HDL	0.0002	0.6035	−0.2069	0.8478
Major depression	0.0317	0.4179	0.0661	0.4524
SBP treatment	−1.6508	13.442	1.1665	23.337
Treated SBP	0.3376	2.7863	−0.2520	4.8378
Average C-statistics	−0.0162	0.0324	−0.0245	0.0491

Abbreviations as in Tables 1, 2, 3, and 4.

Table 6 presents calculated 10-year ASCVD risk scores in Asian and AI women service members given levels of predictors. The new VA women ASCVD risk model estimated 10-year ASCVD risk for Asian and AI service members at 3.30% and 4.32%, respectively, at age 38 years with total cholesterol 190 mg/dL, HDL-C 50 mg/dL, untreated SBP 120 mm Hg, no DM, no current smoking, and no major depression. At age 50 years, the estimated ASCVD risk for Asian and AI service members increased to 6.68% and 8.48% at the same levels of predictors, respectively.

**Table 6:** Ten-Year ASCVD Event Risk in Asian/AI Women: Part 1

Age, y	Asian		AI	
S(10)^a^	0.9729124		0.9628521	
	ASCVD Risk, %^b^^,^^c^	95% CI^d^	ASCVD Risk, %^b^^,^^c^	95% CI^d^
38	3.30 (3.87)	3.12-3.36 (3.19-5.04)	4.32% (4.57)	4.00-4.47 (3.34-5.98)
50	6.68 (8.25)	5.81-7.50 (6.26-10.60)	8.48% (8.96)	6.74-10.23 (6.18-12.42)

Abbreviations as in Tables 1, 2, 3, and 4.

a S (·) is the survival probability function and S(10) is the 10-year ASCVD event-free survival probability. Risk was calculated following: 1- S(10)·exp (Χ β-$\bar{Χ}$ β), where *x* is a vector of covariates in the model and $\bar{Χ}$ is the mean value of corresponding covariates, and β is a vector of risk coefficient corresponding covariates, *x*, at age 38 and 50 years.

b Specific values of *x* are total cholesterol 190 mg/dL, HDL 50 mg/dL, untreated SBP 120 mm Hg, no DM, no current smoking status, and no major depression. In parentheses are the risks when major depressive symptoms are present.

c ASCVD events are the first incidences of any events of nonfatal myocardial infarction, nonfatal stroke, nonfatal cardiac arrests, and cardiac death.

d A 95% CI of the predicted 10-year ASCVD risk score is calculated using a formula, risk score ± 1.96 · SE (risk score), where SE (risk score) = sqrt(x ⋅ cov($\hat{\beta }$ ˆ) ⋅ x^T^), where cov($\hat{\beta }$) is a variance-covariance matrix of estimated coefficients ($\hat{\beta }\text{,}$Supplemental Tables 1 and 2 for Asian and AI women, respectively), *x* is a column vector of all covariates (9x1), and *x*^T^ a transpose of a vector of x; 1.96 is a z score of a standard normal distribution at α = 0.05 for a 2-sided test.

Adjusted to different levels of predictors—total cholesterol 213 mg/dL, HDL-C 54 mg/dL, untreated SBP 136 mm Hg, no DM, no current smoking status, and no major depression—the new VA women CVD risk scores calculate 10-year ASCVD risk scores for Asian and AI women service members aged 46 years as 5.74% and 7.63%, respectively, and at age 55 years as 9.08 % and 11.74%, respectively (Table 7). With presence of depressive symptoms, the estimated 10-year ASCVD risk, numbers in parentheses represent estimated risk when major depression is present, increased by 0.25% to 2.11% and (Tables 6 and 7).

**Table 7:** 10-Year ASCVD Event Risk in Asian/AI Women: Part II

Age, y S(10)^a^	Asian		AI	
	0.9729124		0.9628521	
	ASCVD Risk, %^b^^,^^c^	95% CI^d^	ASCVD Risk, %^b^^,^^c^	95% CI^d^
46	5.74 (7.12)	4.49-6.49 (5.03-8.87)	7.63 (8.05)	5.82-10.33 (5.45-12.24)
55	9.08 (11.19)	6.91-10.54 (7.76-14.22)	11.74 (12.38)	8.77-16.18 (8.28-18.94)

Abbreviations as in Tables 1, 2, 3, and 4.

a S (·) is the survival probability function and S(10) is the 10-year ASCVD event-free survival probability. Risk was calculated following: 1− S(10)·exp $(Χ\hat{\beta }$-$\bar{Χ}\hat{\beta }$), where *x* is a vector of covariates in the model and $\bar{Χ}$ is the mean value of corresponding covariates, and $\hat{\beta }$ is a vector of risk coefficient corresponding covariates, *x*, at age 46 and 55 years.

b Specific values of *x* are total cholesterol 213 mg/dL, HDL 54 mg/dL, SBP 138 mm Hg, no DM, no current smoking status, and no major depression. In parentheses are the risks when major depressive symptoms are present.

c ASCVD events are the first incidences of any events of nonfatal myocardial infarction, nonfatal stroke, nonfatal cardiac arrests, and cardiac death.

d A 95% CI of the predicted 10-year ASCVD risk score is calculated using a formula, risk score ± 1.96 · SE (risk score) where SE (risk score) = sqrt(x ⋅ cov($\hat{\beta }$ ˆ) ⋅ x^T^), where cov($\hat{\beta }$) is a variance-covariance matrix of estimated coefficients ($\hat{\beta }\text{,}$Supplemental Tables 1 and 2 for Asian and AI women, respectively), *x* is a column vector of all covariates (9x1), and *x*^T^ a transpose of a vector of *x*; 1.96 is a *z* score of a standard normal distribution at α = 0.05 for a 2-sided test.

## Discussion

Overall, the new VA women CVD risk scores accurately estimate 10-year ASCVD risk for both Asian and AI women service members and veterans aged 20 to 80 years old with good discrimination (C-statistics >0.77) and accuracy (calibration plots approximation to a 45-degree line) and are well internally validated (bias close to 0 and root mean squared error <4) (Central Illustration).

To date, these are the first validated 10-year ASCVD scores tailored for Asian and AI women service members and veterans. Variances in baseline risk factors for Asian and AI women—greater smoking, diabetes, depression, and antihypertensive medication in AI—can be partly accounted for by sociocultural factors, such as historical trauma and enculturation.^28^ Asian American women differ from N-H White, N-H Black, and Hispanic women in the underlying causes of increased CVD risk,^29^ one of which is social determinants, such as language barriers, immigration status, health illiteracy, and lack of health insurance.^30^ In addition, Asian American individuals are composed of ethnically and culturally diverse sub-ethnic groups, including Chinese, Filipino, Indian, Japanese, and Korean ethnic groups, as well as Hawaiian American. Each sub-ethnic group exhibits different CVD risk, thus categorizing all the diverse ethnic and cultural sub-ethnic groups into 1 Asian race group and creating 1 specific CVD risk score for this group is challenging.^31^ The current study addresses this gap and the methodological challenges in managing the CVD risk heterogeneity among Asian sub-ethnic groups in constructing ASCVD risk scores by using EHR data from Asian women military service members who share commonalities (ie, military exposure) and have equal access to health care via military and VA health systems, to some extent. For example, for Gulf War veterans, environmental exposure to certain toxicants and hazards is found to be associated with ASCVD.^4^ The new Asian VA women CVD risk score is the first validated ASCVD risk score specific to Asian women service members and veterans and was created using their own EHR data as a development cohort and is, thus, appropriate to assess a 10-year ASCVD risk score of Asian women service members aged 20 to 80 years.

A nonlinear aging trajectory in ASCVD risk has been noted by the new Asian and AI VA women CVD risk scores. ASCVD risk in Asian women service members has been accelerating starting from age 46 years, assuming no change in levels of risk factors, and by the time they reach age 50, the risk is estimated to be significantly elevated (Tables 6 and 7). The new Asian VA women CVD risk score can serve as a clinical decision support tool for CVD assessment and treatment, thereby improving CVD care delivery and preventing ASCVD events in Asian women service member and veteran patients at military and VA health care settings.

Despite increasing efforts in improving cardiovascular health in the AI population, the ASCVD burden within this population remains high.^32^^,^^33^ The current available traditional ASCVD risk scores, such as the AHA’s ASCVD scores,^16^^,^^17^ severely underestimate risk in the AI population because of the lack of inclusion of AI-specific data in a development cohort. The current study addresses this gap by leveraging the large-scale VA and military health EHR data, which include a substantially large number of AI women.

Approximately 16,000 AI women veterans were enrolled in VA benefits in 2017, and the proportion of AI women veterans was significantly higher than that of women veterans of other races, according to the VA report “American Indian and Alaska Native Veterans: 2017.”^34^

There is an existing risk score that estimates 10-year ASCVD risk of AI women, using data from the Strong Heart Study, whose development cohort consisted of civilian AI women.^35^ However, AI women service members’ risk score in the Strong Heart Study is much lower by about 2% to 7% compared with the new AI VA women CVD risk score (Supplemental Table 3). The study furthermore calculated the NRI closely following Pencina et al^26^ and Jeon-Slaughter et al^10^^,^^19^ using the study’s development cohort of AI women military service members and found the NRI of the study risk score of 0.795 with 95% CI 0.653-0.829 against the Strong Heart Study risk calculator. The NRI is one of the statistics that compares 2 different risk prediction models and evaluates model performance in prediction accuracy. The positive value of the NRI suggests a better predictability of the current VA women CVD risk score than the existing Strong Heart Study risk calculator in estimating a 10-year ASCVD risk in AI women military service members. A higher ASCVD risk in women military service members and veterans is aligned with clinical assessment because women service members have a greater number of CVD risk factors^2^ and a higher CVD-related mortality risk than their civilian counterparts due to military exposure.^1^ Thus, the development of the new VA AI women CVD risk score from AI women service members’ own EHR data is highly relevant to the current demand for a clinical decision support tool to provide timely preventive interventions and better CVD care for AI women service members and veterans. This will also lead to the potential to improve long-term health outcomes for these groups.

Furthermore, the VA women CVD risk score includes major depression in calculating 10-year ASCVD risk, and its effect amplifies with older age. Holding other risk factors constant, depression, in both Asian and AI women veterans and service members, increased ASCVD risk, and is in line with prior research, especially in women.^36^ Major depression was added as a new independent risk factor predicting increasing ASCVD risk in the VA women risk score for Asian and AI women military members. Asian and AI women military members tend to have poorer mental health and were less likely to use mental health services than their other race counterparts.^37^

With presence of major depression, Asian women service members at age 46 years with untreated SBP 120 mm Hg, total cholesterol 190, HDL 50, no diabetes, and no current smoking will be at a moderate risk for treatment consideration, whereas without considering major depressive symptoms their risk will be reduced to the low-risk category. The new VA Asian women CVD risk score is more sensitive in major depression than the new VA AI women CVD risk score. Major depression is more prevalent among aging Asian women and is undertreated because of mental health stigma, intergenerational cultural conflict in the family, and the experience of racial discrimination.^38^^,^^39^ Integrated care approaches that incorporate mental health services within primary care and cardiology settings could facilitate better management of these comorbidities. Overall, women military service members and veterans are at high risk of major depression,^40^^,^^41^ and the new VA Asian women CVD risk score can serve as a point-of-care tool for Asian women military service members, which will lead to better CVD and overall care for Asian military service members.

### Study limitations and future directions

Despite the strengths of the study, a few limitations are noted. The VA AI women CVD risk score may still underestimate 10-year ASCVD risk in AI women service members due to underreporting of AI race. In addition, the new VA Asian women CVD risk score may inaccurately, over- or underestimate for specific Asian sub-ethnic groups by combining all Asian women into 1 category. However, observed or reported difference in overall ASCVD risk among Asian civilian sub-ethnic groups (eg, South Asian,^12^ Filipino, and Hawaiian American)^42^ can often be attributed to cultural differences and shared environmental exposure. These factors may be offset in the military context due to shared military exposure and equal access to health care through military health and VA settings for women service members and veterans. Therefore, it is justified to develop a single VA Asian women CVD risk score for all Asian women service members and veterans, rather than further stratifying into each Asian sub-ethnic group. Although the VA Asian and AI women CVD risk scores are validated for the U.S. military population, use of the scores in the nonmilitary population (ie, civilians) or Asian countries requires external validation using data from the nonmilitary population. Future research should compare the VA women CVD risk score with other risk scores, such as the Strong Heart Study risk score in AI civilians, and examine ASCVD risk in Asian sub-ethnic groups, especially in civilian populations. The model may need a temporal validation when the new data 2022-2032 are available for a contemporary update of the VA women CVD risk score. Implementation of the new Asian and AI VA women CVD risk score at the VA EHR system would require education, ease of access, and usability and physicians’ adoption of the new score may be slow. Last, although ICD codes are reliable and valid measures to identify ASCVD events, future research also should examine additional measures to prevent potential misclassification bias.^43^

## Conclusions

The new VA Asian and AI women CVD risk scores are the first internally validated 10-year ASCVD risk calculators that can be used as clinical decision support tools to accurately assess CVD risk in the ever-growing Asian and AI women service member population in the VA health care setting.

## Funding Support and Author Disclosures

The study was funded by the U.S. Department of Defense Peer-Reviewed Medical Research Investigator Initiated Award (W81XWH2211018); Dr Haekyung Jeon-Slaughter is the study's principal investigator. The views, opinions, and/or findings contained in this article are those of the authors and should not be construed as an official Department of the Army position, policy, or decision unless so designated by other documentation. The authors have reported that they have no relationships relevant to the contents of this paper to disclose.
