# Supplementary material for: New 10-Year Atherosclerosis Cardiovascular Disease Risk Score for Asian and American Indian Women Service Members
Source: JACC Asia. 2026 Feb 27;6(8):1765–76. doi: 10.1016/j.jacasi.2026.01.003 (PMC13491086; doi:10.1016/j.jacasi.2026.01.003)
Supplement: Supplementary file 1 [file mmc1.docx]

A. **Supplemental methods**
1. **Schoenfeld plots**

Proportional hazard assumptions for Cox models were tested for all covariates using Schoenfeld residual plots. The residual plots were close to “0” over the time for all covariates and Schoenfeld residual tests failed to reject Proportional hazard assumptions for both Asian and American Indian (AI) VA women CVD models. See supplementary Figures 1 and 2.

2. **Data construct and missing data**

The study constructed longitudinal data of the VA women cohort with all available person‐level visits for application of a time‐variant covariate Cox model.

First, the study consolidated all visit date longitudinal data into month long intervals during the entire study period of January 1, 2007 to December 31, 2022. Each record then represented a visit with a month interval. For example, the baseline visit was defined as the first month interval, January 1, 2007, and the next available outpatient or inpatient visit as the next visit, and so forth. If there were multiple visit records within the month, then visit records were consolidated into one data point per month visit. Multiple visit records of dichotomized risk factors, such as DM, current smoking status, antihypertensive medication intake, and any CVD events, were consolidated into a maximum value of 1 (yes) and 0 (no). For continuous risk factors such as systolic blood pressure (SBP), total cholesterol, and high‐density lipoprotein cholesterol (HDL‐C), the average values of the factors over the multiple visit records were selected as a visit data point. The first occurrence of any CVD events was an endpoint and when the patient died from diseases or causes other than cardiovascular diseases, the follow‐up data of the patient were left as missing (i.e., censored).

Second, the longitudinal data were reconstructed to fit the time‐varying covariate Cox model estimation. The second step created time intervals between 2 consecutive nonmissing visits per patient. The number and length of the time intervals for each patient reflected the number of available visits and their durations. The first time interval was set as the baseline. The current study assumed no loss to follow‐up; thus, the last time interval was set to be either a visit of death occurrence, or the last visit of the study period. Inherent to EHR data, unequal visit‐to‐visit intervals per patient were created which is partly due to different visit dates and intervals on EHR data collection on each covariate. Lipid panels and SBP data were collected at different visits and unequal visit intervals. SBP were collected every visit while lipid panels data are not—6 months to >3 year intervals and results are often delayed or lagged much longer than one month after being ordered. Constructing all variables, which were collected at different visits, into one single longitudinal data per patient creates empty cells of covariate data when the covariates were not collected at the same visit. Empty cells of SBP and cholesterol data were filled with last observed values until the new values observed. Thus, each patient needs at least one data point of continuous covariates such as SBP and cholesterol data. For dichotomized covariates, DM, major depression, and current smoking status, the last observation move forward was used up to one year follow-up. The study defined 6 months as a minimum interval length to capture time variance in factors such as refills of antihypertensive medications.^20^

3. **Variable Construct**

CVD events, DM, and major depression study variables were constructed using International Classification of Diseases (ICD), Ninth Revision (ICD‐9) and Tenth Revision (ICD‐10) diagnosis and procedure codes extracted from the VA EHR data. DM was created not only using ICD coded but also used HbA1C cut off value ≥ 6.5% (48mmol/mol) and we used within 6 month medication dispense date of antihypertensive medication.^10,20^ Current smokers were defined using health factors (see Chen et al. 2020).^20^ The ICD codes to define each variable were validated using available documentations from the VA Phenomics Library. The VA Phenomics Library compiled VA EHR‐based phenotype algorithms including ICD codes to define diseases. Chen et al.^20^ described detailed ICD codes used to construct each risk factor included in the CVD risk score calculation and validation of ICD codes used to construct CVD events with providers' notes. The study followed Chen et al., 2020^20^ and Jeon-Slaughter et al., 2021^10^ closely for missing data and its imputation.

Due to inherent characteristics of EHR records, there exist multiple visit records with

different dates of vital signs and laboratory results (such as lipid panel results) and medication records per patient and some of these records have incomplete data. Our study strategy to deal with this issue of EHR records was not to impute incomplete data on vital signs such as blood pressure and cholesterol values when they are not available during the study period. Under this strategy, we defined VA women as those who were actually treated at VA health care system by confirming visit records with vital sign data, Blood pressure data, recorded and entered during the visit. The VA standard treatment procedure guideline for VA outpatient and inpatient visits is to measure, record, and enter blood pressure values into medical records. Thus, if blood pressure data are missing from the visit records, we can assume the patient may not have been treated at VA health care system.

For missing data on cholesterol data, we restricted our study cohort to those patients with

complete cholesterol data since the ACC/AHA model structure is following the cholesterol model and having complete cholesterol is essential for ACC/AHA model. When either total cholesterol or High Density Lipoprotein (HDL) cholesterol was missing but other lipid panel test results such as Low Density Lipoprotein (LDL)-cholesterol and triglyceride were available from a lipid panel test and units, mg/dL and mmol/L, we then calculated them by applying a formula, total cholesterol= HDL + LDL + 1/5 triglyceride.

4. **Model Estimation**

Stratified by race and ethnicity, the new Asian/ AI VA women CVD risk model estimated risk coefficients and 10‐year ASCVD risk using a time‐variant covariate Cox model. The factors included in the model were age, untreated and treated SBP (in millimeters of mercury), presence of DM (yes versus no), current smoking status (yes versus no), total cholesterol (in milligrams per deciliter), HDL‐C (in milligrams per deciliter), and presence of major depressive episodes/diagnosis (yes versus no). Treated SBP was defined as an interaction between on antihypertensive medication and SBP. We introduced an interaction term to include untreated and treated SBP in the model. We also added a variable of antihypertensive medication indicator as treated, unlike other prediction model we have a full interaction term with SBP-- antihypertensive medication treatment + antihypertensive medication treatment* SBP (antihypertensive medication treatment = 0 (no) or 1 (yes). It is typical to see a big SE for an indicator in interaction terms. We have utilized penalized Cox model to test an inclusion of interaction term.

Data on factors were repeatedly measured from multiple visits during the study period. Age, SBP, total cholesterol, and HDL‐C were natural log transformed to follow log normal distributions. Each VA woman had CVD risk factors measured repeatedly from multiple visits during the study period. The time‐variant covariate Cox model assumes that effects of time‐varying factors on CVD risk follow step functions.

Before determining the final model construct of the VA women CVD risk score, the study team extensively investigated in aging trajectory modeling. Interaction term of age and age non linear function in probability of ASCVD event in 10 years. We explored non linearity of continuous variables by squared and polynomial function see Chen et al. 2020.^17^

The study conducted sensitivity tests for final models by conducting penalized Cox model in including variables of untreated SBP, treated SBP and on antihypertensive medication, interaction terms, Body Mass Index (BMI), LDL, and Diastolic Blood Pressure (DBP), following model fit induces and tests such as log likelihood tests and Akaike Information Criteria (AIC) for multicollinearity.

5. **Internal and cross validation**

For Asian women military service members, number of mean events was 519 for training data sets and 52 for testing data sets.

For AI women military service members, number of mean events was 263 for training data sets and 25 for test data sets.

**Supplemental Table 1. Asian Veteran Affairs (VA) Women Cardiovascular Disease (CVD) Risk Score Variance and covariance matrix estimates cov(**$\hat{\beta}$**)**

|  | Ln Age | Ln SBP | DM | Current Smoker | Ln Total Cholesterol | Ln HDL | Major Depression | SBP Treatment | Treated SBP |
| --- | --- | --- | --- | --- | --- | --- | --- | --- | --- |
| Ln Age | 0.046 | -0.023 | -0.006 | 0.004 | 0.014 | 0.0003 | 0.003 | 0.021 | -0.005 |
| Ln SBP | -0.023 | 0.418 | 0.002 | 0.005 | -0.014 | 0.018 | -0.005 | 1.938 | -0.403 |
| DM | -0.006 | 0.002 | 0.016 | -0.001 | 0.005 | 0.002 | 0.0004 | -0.012 | 0.002 |
| Current Smoker | 0.004 | 0.005 | -0.0006 | 0.028 | -0.003 | 0.004 | -0.0004 | 0.021 | -0.004 |
| Ln Total Cholesterol | -0.014 | -0.013 | 0.005 | -0.003 | 0.075 | -0.009 | -0.002 | -0.006 | 0.001 |
| Ln HDL | 0.0003 | 0.018 | 0.002 | 0.004 | -0.009 | 0.031 | 0.002 | 0.093 | -0.018 |
| Major Depression | 0.003 | -0.005 | 0.0004 | -0.0004 | -0.002 | 0.002 | 0.014 | -0.046 | 0.009 |
| SBP Treatment | 0.021 | 1.938 | -0.012 | 0.021 | -0.007 | 0.092 | -0.047 | 19.368 | -3.995 |
| Treated SBP | -0.005 | -0.403 | 0.002 | -0.004 | 0.001 | -0.019 | 0.009 | -3.995 | 0.825 |

Abbreviations. DM = Diabetes Mellitus; HDL = High Density Lipoprotein Cholesterol; Ln = Natural log; SBP = Systolic Blood Pressure

**Supplemental Table 2. American Indian (AI) Veterans Affairs (VA) Women Cardiovascular Disease (CVD) Risk Score Variance and covariance matrix estimates cov(**$\hat{\beta}$**)**

|  | Ln Age | Ln SBP | DM | Current Smoker | Ln Total Cholesterol | Ln HDL | Major Depression | SBP Treatment | Treated SBP |
| --- | --- | --- | --- | --- | --- | --- | --- | --- | --- |
| Ln Age | 0.122 | -0.178 | -0.015 | -0.008 | -0.036 | 0.0002 | 0.0001 | -6.355 | 0.129 |
| Ln SBP | -0.178 | 1.714 | 0.012 | 0.007 | 0.005 | 0.094 | 0.005 | 7.888 | -1.634 |
| DM | -0.015 | 0.012 | 0.035 | 0.008 | 0.007 | 0.008 | 0.002 | 0.0002 | -0.001 |
| Current Smoker | -0.008 | 0.007 | 0.008 | 0.038 | 0.007 | 0.002 | -0.001 | -0.190 | 0.039 |
| Ln Total Cholesterol | -0.036 | 0.005 | 0.007 | 0.007 | 0.145 | -0.026 | 0.007 | 0. 106 | -0.022 |
| Ln HDL | 0.0002 | 0.094 | 0.008 | 0.002 | -0.026 | 0.105 | 0.009 | 0. 293 | -0.059 |
| Major Depression | 0.0001 | 0.006 | 0.002 | -0.001 | -0.007 | 0.009 | 0.029 | -0.174 | 0.035 |
| SBP Treatment | -0.635 | 7.888 | 0.0002 | -0.190 | 0.106 | 0.293 | -0.174 | 0. 607 | -12.551 |
| Treated SBP | 0.129 | -1.634 | -0.001 | 0.039 | -0.022 | -0.059 | 0.035 | -0.125 | 2.595 |

Abbreviations. DM = Diabetes Mellitus; HDL = High Density Lipoprotein Cholesterol; Ln = Natural log; SBP = Systolic Blood Pressure

**Supplemental Table 3. Strong Heart Study risk score calculator: Ten‐Year ASCVD Risk for AI women service members and veterans**

| Age | ASCVD risk (%) |
| --- | --- |
| 38 yrs | 2.0%^1^ |
| 50 yrs | 4.0%^1^ |
| 46 yrs | 4.0%^2^ |
| 55 years | 6.0%^2^ |

Abbreviations. AI = American Indians or Alaska Natives; ASCVD = Atherosclerosis Cardiovascular Disease; CVD = Cardiovascular Disease; VA = Veterans Affairs

Notes. 1. Specific values of factors are total cholesterol 190 mg/dL, high‐density lipoprotein 50 mg/dL, untreated systolic blood pressure 120 mm Hg, no diabetes mellitus, no current smoking status, and no major depression.

2. Specific values of factors are total cholesterol 213 mg/dL, high‐density lipoprotein 54 mg/dL, untreated systolic blood pressure 138 mm Hg, no diabetes mellitus, no current smoking status, and no major depression.

3. Strong heart study risk calculator is available at https://strongheartstudy.org/Community/Risk-Calculators

**Supplemental Figure 1. Schoenfeld residual plots for time-varying covariates: Asian women military service members**

Asian = Asian Americans or Hawaiian Americans; HDL = High Density Lipoprotein; Ln = natural logarithm; SBP = Systolic Blood Pressure

**Supplemental Figure 2. Schoenfeld residual plots for time-varying covariates: American Indians and Alaska Natives (AI) women military service members**

HDL = High Density Lipoprotein; Ln = natural logarithm; SBP = Systolic Blood Pressure
